# The First-Trimester Gestational Weight Gain Associated With *de novo* Hypertensive Disorders During Pregnancy: Mediated by Mean Arterial Pressure

**DOI:** 10.3389/fnut.2022.862323

**Published:** 2022-04-13

**Authors:** Zhichao Yuan, Hai-Jun Wang, Tao Su, Jie Yang, Junjun Chen, Yuanzhou Peng, Shuang Zhou, Heling Bao, Shusheng Luo, Hui Wang, Jue Liu, Na Han, Yuelong Ji

**Affiliations:** ^1^Department of Maternal and Child Health, School of Public Health, Peking University, Beijing, China; ^2^National Health Commission Key Laboratory of Reproductive Health, Beijing, China; ^3^Maternal and Child Health Care Hospital of Tongzhou District, Beijing, China; ^4^Department of Electrical and Computer Engineering, Whiting School of Engineering, Johns Hopkins University, Baltimore, MD, United States; ^5^Department of Epidemiology and Biostatistics, School of Public Health, Peking University, Beijing, China

**Keywords:** *de novo* hypertensive disorders of pregnancy, gestational weight gain, mean arterial pressure, overweight, obesity, national academy of medicine criteria

## Abstract

The relationship between first-trimester GWG (_*T*1_GWG) and risk of hypertensive disorders of pregnancy (HDP) remained uncertain. This study aimed to investigate the association between _*T*1_GWG and risk of *de novo* HDP. Meanwhile, we explored the mediated effect and constructed an early GWG category to evaluate the predictive capacity for HDP. _*T*1_GWG was defined as the weight difference between 13 ± 1 gestational weeks and pre-conception. HDP group was defined as having diagnosis of *de novo* HDP, including gestational hypertension or *de novo* pre-eclampsia (PE) during the current pregnancy. Early GWG category was constructed according to the risk of HDP within each pre-pregnancy body mass index (BMI) group. Cox regression model was utilized to check the association between the _*T*1_GWG and HDP. Serial mediation model was adopted to evaluate the potential mediators including mean arterial pressure (MAP) at 13th and 20th week. The logistic regression model with bootstrap was performed to assess the predictive capacity of Early GWG category and MAP for the risk of HDP. A total of 17,901 pregnant women (mean age, 29.0 years) were recruited from 2013 to 2017 at the Tongzhou Maternal and Child Health Hospital in Beijing, China. Compared to women in Class 1 of early GWG category, women in the Class 2, 3, 4 have increased risks of HDP by 1.42, 4.27, and 4.62 times, respectively (hazard ratio [*HR*] = 2.42, 95% *CI*: 2.11–2.77; *HR* = 5.27, 95% *CI*: 4.05–6.86; *HR* = 5.62, 95% *CI*: 4.05–7.79). The MAP measured at 13th and 20th week totally mediated 33.1 and 26.7% of association between _*T*1_GWG GWG and HDP in total participants and overweight/obesity pregnancies, respectively. The area under receiver operator characteristic curve for predictive model utilizing early GWG category and MAP measured at 13th and 20th week for the risk of HDP is 0.760 (95% *CI*: 0.739–0.777). The _*T*1_GWG was associated with *de novo* HDP, which was partially mediated by MAP measured at 13th and 20th week. Early GWG category showed a better predictive capacity for the risk of HDP compared to the National Academy of Medicine criteria for _*T*1_GWG.

## Introduction

Hypertensive disorders of pregnancy (HDP) are characterized by the abnormal elevation of blood pressure during pregnancy. It can be classified into four categories: chronic hypertension, gestational hypertension (GH), *de novo* and superimposed pre-eclampsia (PE), and eclampsia (1). GH and *de novo* PE are regarded as *de novo* HDP. Mean arterial pressure (MAP) was consisted of systolic blood pressure (SBP) and diastolic blood pressure (DBP) as a composite index to assess the blood pressure in clinical practice. Previous studies have inducted that the blood pressure was an important predictive factor to evaluate the risk of HDP before diagnosis (2, 3). The HDP are the leading causes of maternal and fetal morbidity and mortality globally. HDP is a dominant cause of maternal death in developing countries, accounting for one fifth of maternal deaths worldwide (4). The etiology of *de novo* HDP is still unknown. A recent review summarized the main modifiable risk factors, such as body mass index, anemia, lower education level; and non-modifiable risk factors, such as maternal age, primiparous, multiple pregnancy, HDP history, gestational diabetes mellitus, pre-existing type 2 diabetes mellitus, pre-existing urinary tract infection, single nucleotide polymorphism in the angiotensinogen gene, and a family history of HDP or type 2 diabetes mellitus (5).

Gestational weight gain (GWG) is a natural process during pregnancy. The change in maternal lifestyle and fetus, such as increasing nutrient intake, decreasing physical activity, and fetal growth, will both lead pregnant women to increase their body weight. Deviations from proper weight gain in either direction during pregnancy have been found to be associated with adverse pregnancy outcomes, such as small for gestational age (SGA), large for gestational age (LGA), macrosomia, cesarean delivery, gestational diabetes mellitus, PE, postpartum weight retention, and obesity of the offspring (6). The guideline for GWG from the National Academy of Medicine (NAM) have been applied to guide pregnancy weight management for over 30 years in the United States (7). The updated NAM guideline in 2009 provided a body mass index (BMI)-specific guideline (8), which provides a major set of recommendations for optimal total GWG during the entire pregnancy. HDP, usually diagnosed in the second and third trimester, was found to associate with the total GWG by several studies (9–11). Since the total GWG is calculated based on the subtraction between the pre-pregnancy and antepartum weights, the association between total GWG and HDP published previously could not provide evidence for a temporal relationship.

Gestational weight gain as a non-intrusive and easily attainable clinical indicator has been applied to estimate the risk of adverse complications, such as GDM, SGA, and LGA. Due to the absence of temporal relationship, previous studies focusing on total GWG and HDP cannot provide risk assessment of HDP in terms of GWG. This study aimed to assess the association between first-trimester GWG (_*T*1_GWG) and *de novo* HDP. Meanwhile we explored the mediators and evaluated the predictive capacity of _*T*1_GWG and mediators for HDP.

## Materials and Methods

### Study Design and Participants

**FIGURE 1:**
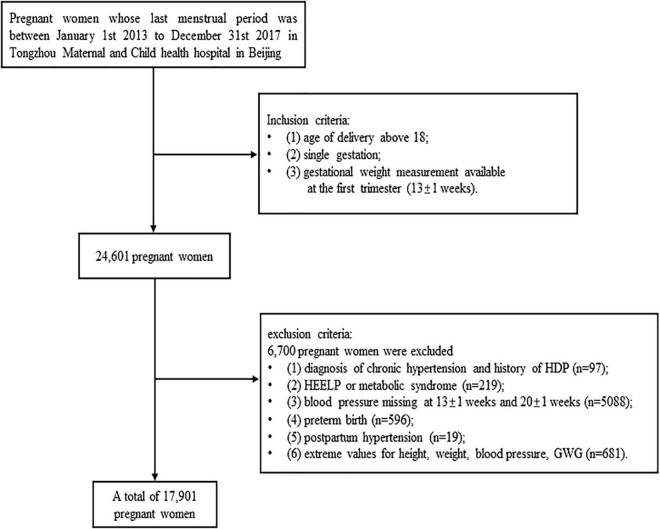
Study flowchart. Flowchart illustrating the selection of the study population in current study.

This study analyzed the data of a birth cohort, which were collected from 2013 to 2017 at Tongzhou Maternal and Child health hospital in Beijing, China. There are 17,901 pregnant women enrolled in the current study. The inclusion criteria of this cohort study were: (1) age of delivery above 18 years, (2) single gestation, (3) _*T*1_GWG available at the first trimester (13 ± 1 weeks). The exclusive criteria were: (1) diagnosis of chronic hypertension and history of HDP, (2) HEELP or metabolic syndrome, (3) blood pressure missing at 13 ± 1 and 20 ± 1 weeks, (4) preterm birth, (5) postpartum hypertension, (6) extreme values for height, weight, and blood pressure (height < 1 m or height > 2 m, weight < 30 kg or weight > 150 kg, SBP < 70 mmHg or SBP > 270 mmHg, DBP < 50 mmHg or DBP > 140 mmHg, _*T*1_GWG < −5.9 kg or _*T*1_GWG > 11 kg). The flow chart was presented as Figure 1.

### Data Collection

The sociodemographic data of pregnant women were collected from the first antenatal clinical record, such as race, age, level of education, employment condition, elder gestation, gestational season, elder gestation, and pre-pregnant weight and height. The pre-pregnant weight was obtained from the pregnant women’s self-reports. The gestational weight and blood pressure were measured by trained nurses at regular antenatal clinic. The GWG was defined as the difference between gestational weight and pre-pregnant weight. The MAP equaled to SBP plus 2/3 DBP. The pre-pregnant BMI was calculated using formula: BMI = (weight in kg) divided by (square of height in m). The pre-pregnant BMI class was identified according to the BMI criteria for Asian women (the BMI criteria of underweight, normal weight, overweight, and obesity were defined as < 18.5, 18.5–23, 23–27.5, ≥27.5) (12). *De novo* HDP that includes GH and *de novo* PE was the primary outcome of this study. The diagnosis of GH or *de novo* PE was made by obstetricians according to the latest Chinese clinical practice guideline which was consistent with the diagnostic guidelines in the developed countries (13). International Classification of Diseases 10 (ICD-10) codes were used to define GH and *de novo* PE (such as O13.01, O13.02 for GH; O11.01, O14.001, O14.101, O14.102, O14.901 for *de novo* PE). The text containing information related to GH and *de novo* PE in medical records was extracted to double confirm the disease and identify the week of disease diagnosis. The normotensive group was defined as free from any diagnosis of GH, PE, and without a history of hypertensive disorders.

### Ethics

The study was approved by the Institutional Review Board of Peking University Health Science Center (No. IRB00001052-21023).

### Statistics

Missing values of demographic variables, we imputed the missing data by the k-nearest neighbor (KNN) algorithm [Beretta and Santaniello (14)]. The Shapiro–Wilk test was applied to check the normality of the distribution for each variable. Normally distributed variables were presented as means with SD and analyzed with *t*-test for group comparisons between HDP and Normotensive groups. Non-normally distributed variables were shown as medians with interquartile range and analyzed with chi-square test or Mann–Whitney U-test.

An early GWG category (EwtGCat) in terms of the risk of HDP was constructed. The risk ratios (RR) for HDP were calculated using the multivariable Poisson regression model for each _*T*1_GWG interval within the particular pre-pregnant BMI class *vs*. all other women within that BMI class, borrowing the LifeCycle Project method (6). Considering the _*T*1_GWG was in a relatively small scale, we choose the 25th, 50th, 75th percentiles as the cut-off points for the category. The Class 1 of the EwtGCat included the women with underweight and normal weight pre-pregnant BMI. The women with overweight or obese during pregnancy were further grouped into class 2, 3, and 4. Class 2 was defined as the weight gain with significant negative association (*RR* < 1). Class 3 was the weight gain interval without statistical difference. Class 4 was the weight gain interval that showed remarkable positive association (*RR* > 1). The continuous _*T*1_GWG was also transformed into categorical variables using quartile method and NAM criteria. Multivariable Cox hazards regression model was utilized (15) to access the association between _*T*1_GWG (continuous, quartile, EwtGCat, and NAM criteria) and HDP after adjusting the covariables. The covariables include race, age, level of education, employment condition, elder gestation, and gestational season.

**FIGURE 2:**
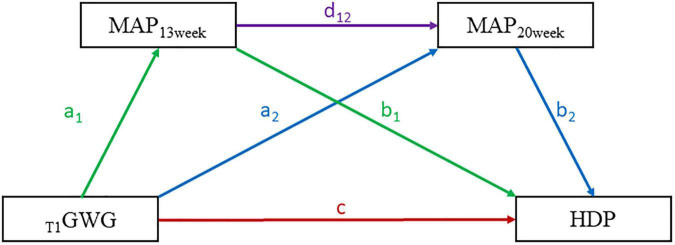
The serial mediation pattern of the association between first-trimester GWG (_*T1*_GWG) and hypertensive disorders of pregnancy (HDP). The respective colorful lines represent the different pathways of the _*T1*_GWG effects on HDP. The direct effect (DI) of _*T1*_GWG on HDP was presented by red line. The indirect effect (IE) of _*T1*_GWG to MAP_13 *week*_ to HDP was presented by green lines. The IE from _*T1*_GWG to MAP_13 *week*_ to MAP_20 *week*_ to HDP was presented by purple lines. The IE of _*T1*_GWG to MAP_20 *week*_ to HDP was presented by blue lines. The serial mediation equation included three parts: (1). MAP_13 *week*_ ∼ a_1_*_T1_GWG + covariables. (2) MAP_20 *week*_ ∼ a_2_*_T1_GWG + d_12_*MAP_13 *week*_ + covariables. (3) HDP ∼ c* _*T1*_GWG + b_1_* MAP_13 *week*_ + b_2_* MAP_20 *week*_ + covariables. And the c was defined as the DI from _*T1*_GWG to HDP, the mediation effects were expressed as follows: (1) IE_1_ (_*T1*_GWG to MAP_13 *week*_ to HDP) = a_1_*b_1_. (2) IE_2_ (_*T1*_GWG to MAP_20 *week*_ to HDP) = a_2_*b_2_. (3) IE_3_ (_*T1*_GWG to MAP_13 *week*_ to MAP_20 *week*_ to HDP) = a_1_*d_12_*b_2_.

We constructed a serial mediation model to assess the effect of _*T*1_GWG on HDP with MAP at 13th and 20th weeks (MAP_13 *week*_ and MAP_20 *week*_) as mediators by adjusting covariables. The direct effect (DE) defined as _*T*1_GWG to HDP. The indirect effects (IE) included three aspects: (1) IE_1_ was defined as the pathway from _*T*1_GWG to MAP_13 *week*_ to HDP, (2) IE_2_ was the pathway from _*T*1_GWG to MAP_20 *week*_ to HDP, (3) IE_3_ was the pathway from _*T*1_GWG to MAP_13 *week*_ to MAP_14 *week*_ to HDP. All the estimated effects were validated by bootstrapping method for 5,000 loops. The serial mediation model pattern was presented as Figure 2. The bruceR package (version 0.7.0) was adopted in this part of analyses. The stratified analyses were conducted to explore the effect of EwtGCat on the risk of HDP across different subgroups, such as parity (unipara *vs*. multipara), maternal age (>35 age *vs*. ≤35 age), conceptional season (spring/winter *vs*. summer/autumn), education level (high *vs*. low). The sensitivity analyses were further performed to assess the association between _*T*1_GWG and onset time of HDP.

The adjusted logistic regression model was adopted to assess the prediction performance for HDP with 1,000 bootstrapping replications (16). Three different models were constructed and compared with each other by the Area Under Receiver Operator Characteristic Curve (AUC), sensitivity, and specificity. The models were as follow: model_*NAM*_ includes NAM criteria and covariables; model_*EwtGCat*_ included EwtGCat and covariables; model_*EwtGCat&MAP*_ included EwtGCat, MAP_13 *week*_, MAP_20 *week*_ and covariables. All the statistical analyses were performed on the R software (version 4.0.0). The *p*-value < 0.05 was considered as significant difference.

## Results

### Characteristics of the Birth Cohort

Table 1 shows the maternal socio-demographical and first-trimester weight gain characteristics between *de novo* HDP and normotensive groups. The percentage of *de novo* HDP in our study was 5.31%. The 17,901 pregnant women included in this study had the mean maternal age of 29.0 years (*SD* = 3.8). Women in the following strata had a significantly higher percentage of HDP diagnosis: educational level with high school or lower, overweight or obesity with pre-pregnant BMI class and unipara. The _*T*1_GWG was significantly higher in HDP group compared with normotensive women (*p* = 0.001). The detailed characteristics of the participants are listed in Table 1.

**TABLE 1:** Maternal socio-demographical and first-trimester weight gain characteristics.

Characteristics	No HDP (*N* = 16950)	HDP (*N* = 951)	*P*-value
_*T*1_GWG, mean(SD)	1.2 (2.4)	1.5 (2.5)	0.001
MAP_13 *week*_, mean (SD)	83.2 (8.2)	88.9 (8.2)	<0.001
MAP_20 *week*_, mean (SD)	80.6 (8.0)	86.9 (8.3)	<0.001
Age, mean(SD)	29.0 (3.8)	29.1 (3.8)	0.800
**Education level, N(%)**			
High school or lower	4269 (25.2)	286 (30.1)	
Vocational college	5440 (32.1)	322 (33.9)	<0.001
University or above	7241 (42.7)	343 (36.1)	
**Employment condition, N(%)**			
Unemployment	2220 (13.1)	128 (13.5)	0.700
Employment	14730 (86.9)	823 (86.5)	
**Race, N(%)**			
Minority	1031 (6.1)	44 (4.6)	0.066
Ethnic Han	15919 (93.9)	803(95.4)	
**Maternal age (>35 age), N(%)**			
No	15432 (91.0)	664 (89.5)	0.100
Yes	1518 (9.0)	189 (10.5)	
**Parity, N(%)**			
Unipara	12447 (73.4)	745 (78.3)	<0.001
Multipara	4503 (26.6)	206 (21.7)	
**Pre-pregnant BMI class, N(%)**			
Underweight	1927 (11.4)	45 (4.7)	<0.001
Normal weight	9596 (56.6)	370 (38.9)	
Overweight	4490 (26.5)	391 (41.1)	
Obesity	937 (5.5)	145 (15.2)	
**Conceptional season, N(%)**			
Spring	4044 (23.9)	311 (32.7)	<0.001
Summer	3696 (21.8)	189 (19.9)	
Autumn	4427 (26.1)	161 (16.9)	
Winter	4783 (28.2)	290 (30.5)	

*_T1_GWG, first trimester gestational weight gain; HDP, hypertensive disorders of pregnancy; BMI, body mass index; N, number; MAP, mean arterial pressure.*

### The Construction of Early Gestational Weight Gain Category

The Early GWG Category (EwtGCat) was constructed according to the Supplementary Table 1. The Class 1 of EwtGCat was identified as the women with underweight and normal weight pre-pregnant BMI classes, and there was no association between _*T*1_GWG and HDP. As for underweight and obesity, the significant negative association was obtained in Class 2 which the interval of _*T*1_GWG was below 0 kg. Class 3 was the interval of _*T*1_GWG from 0 to 2.5 kg which we failed to observe any statistical association. Class 4 was the interval of _*T*1_GWG above 2.5 kg which presented a significant positive association between _*T*1_GWG and HDP.

### The Association Between _*T*1_GWG and *de novo* Hypertensive Disorders of Pregnancy

As the Table 2 shown, _*T*1_GWG was significantly higher in the HDP pregnancies compared with normotensive women (*HR* = 1.07, 95% *CI*: 1.04–1.09, *p* < 0.001). In comparison with women in the lowest quartile of _*T*1_GWG, the hazards of HDP elevated in the women with Q3, Q4 of _*T*1_GWG (*HR* = 1.29, 95% *CI*: 1.08–1.55, *p* = 0.005; *HR* = 1.43, 95% *CI*: 1.21–1.69, *p* < 0.001). There were significant associations between _*T*1_GWG and HDP in the Class 2, 3, 4 compared with Class 1 under EGC criteria (*HR* = 2.42, 95% *CI*: 2.11–2.77, *p* < 0.001; *HR* = 5.27, 95% *CI*: 4.05–6.86, *p* < 0.001; *HR* = 5.62, 95% *CI*: 4.05–7.79, *p* < 0.001). However, there is no significant hazard difference between normal and abnormal groups under NAM criteria (*HR* = 1.04, 95% *CI*: 0.91–1.20, *p* = 0.551).

**TABLE 2:** Hazard ratios for the association between the first trimester gestational weight gain (_*T1*_GWG) and hypertensive disorders of pregnancy (HDP).

	No HDP (N)	HDP (N)	HR	95% CI	*P*-value
_*T*1_GWG (continuous)	16950	951	1.07	1.04–1.09	<0.001
**_*T*1_GWG quartile**					
Q1	6055	298	ref		
Q2	3271	181	1.19	0.99–1.43	0.065
Q3	3510	208	1.29	1.08–1.55	0.005
Q4	4114	264	1.43	1.21–1.69	<0.001
**EwtGCat (category, N)**					
Class 1	11523	415	ref		
Class 2	4916	432	2.42	2.11–2.77	<0.001
Class 3	308	64	5.27	4.05–6.86	<0.001
Class 4	203	40	5.62	4.05–7.79	<0.001
**NAM recommendation (category, N)**					
Normal	11983	666	ref		
Abnormal	4967	285	1.04	0.91–1.20	0.551

*_T1_GWG, the first trimester gestational weight gain; EwtGCat, early gestational weight gain categories; HDP, hypertensive disorders of pregnancy; NAM, national academy of medicine; HR, hazard ratios; CI, confidence interval. The HR was adjusted by race, age, education level, elderly maternal employment condition, parity. The cutoff points of Q1, Q2, Q3, Q4 were defined by the 25th, 50th, 75th quartiles of the distribution of _T1_GWG.*

### The Serial Mediation Effect of Mean Arterial Pressure on the Association Between _*T*1_GWG and Hypertensive Disorders of Pregnancy

The Table 3 indicated that MAP_13 *week*_ and MAP_20 *week*_ totally mediated 37.7% of association between _*T*1_GWG and HDP in all participants (*IE*_*total*_ = 1.001 × 10^–3^, *p* < 0.001; *DE* = 2.654 × 10^–3^, *p* < 0.001). The indirect mediation effect was consisted in 3 pathways, such as (1) _*T*1_GWG to MAP_13 *week*_ to HDP (*IE*_1_ = 3.330 × 10^–4^, mediate proportion: 12.5%, *p* < 0.001); (2) _*T*1_GWG to MAP_20 *week*_ to HDP (*IE*_2_ = 3.970 × 10^–4^, mediate proportion: 15.0%, *p* < 0.001); (3) _*T*1_GWG to MAP_13 *week*_ to MAP_20 *week*_ to HDP (*IE*_3_ = 2.710 × 10^–4^, mediate proportion: 10.2%, *p* < 0.001). We reran the serial mediation model to test the IE of MAP in overweight/obesity women, which is similar to the results of all participants. The MAP_13 *week*_ and MAP_14 *week*_ mediated 26.7% of total effect of _*T*1_GWG on HDP. IE_1_, IE_2_, and IE_3_ explained 6.1, 13.7, and 6.9%, respectively, of the total effect through 3 mediated pathways (all *p* < 0.001). However, we could not detect meaningful mediation effect in the underweight and normal weight women.

**TABLE 3:** Mediation effect of mean arterial pressure (MAP) to the association between the _*T1*_GWG and HDP.

Participant	Mediation effect	Estimate	95% CI	Proportion	*P*-value
**Total participants**					
	Indirect effect	1.001 × 10^–3^	(7.020 × 10^–4^, 1.273 × 10 ^–3^)	37.7%	<0.001
	_*T*1_GWG-MAP_13 *week*_-HDP	3.330 × 10^–4^	(2.060 × 10 ^–4^, 4.880 × 10 ^–4^)	12.5%	<0.001
	_*T*1_GWG -MAP_20 *week*_-HDP	3.970 × 10^–4^	(2.260 × 10 ^–4^, 5.670 × 10 ^–4^)	15.0%	<0.001
	_*T*1_GWG -MAP_13 *week*_-MAP_20 *week*_-HDP	2.710 × 10^–4^	(1.700 × 10 ^–4^, 3.700 × 10 ^–4^)	10.2%	<0.001
	Direct effect				
	_*T*1_GWG -HDP	1.652 × 10^–3^	(1.860 × 10 ^–4^, 3.105 × 10 ^–3^)	62.3%	0.026
	Total effect	2.654 × 10^–3^	(1.157 × 10 ^–3^, 4.113 × 10 ^–3^)	100.0%	<0.001
**Overweight/obesity participants**					
	Indirect effect	1.853 × 10^–3^	(1.233 × 10^–3^, 2.563 × 10^–3^)	26.7%	<0.001
	_*T*1_GWG -MAP_13 *week*_-HDP	4.200 × 10^–4^	(1.780 × 10^–4^, 7.120 × 10^–4^)	6.1%	<0.001
	_*T*1_GWG -MAP_20 *week*_-HDP	9.500 × 10^–4^	(5.630 × 10^–4^, 1.386 × 10^–3^)	13.7%	<0.001
	_*T*1_GWG -MAP_13 *week*_-MAP_20 *week*_-HDP	4.810 × 10^–4^	(2.240 × 10^–4^, 7.680 × 10^–4^)	6.9%	<0.001
	Direct effect				
	_*T*1_GWG -HDP	5.088 × 10^–3^	(2.126 × 10^–3^, 7.987 × 10^–3^)	73.3%	<0.001
	Total effect	6.941 × 10^–3^	(4.035 × 10^–3^, 9.835 × 10^–3^)	100.0%	<0.001

*_T1_GWG, the first trimester gestational weight gain; MAP, mean arterial pressure; HDP, hypertensive disorders of pregnancy. The serial mediation model was adjusted by race, age, level of education, employment condition, maternal age (>35 age), conceptional season. The pattern of serial mediation model was presented in Figure 2.*

### The Subgroup Analyses for the Early Gestational Weight Gain Category

**FIGURE 3:**
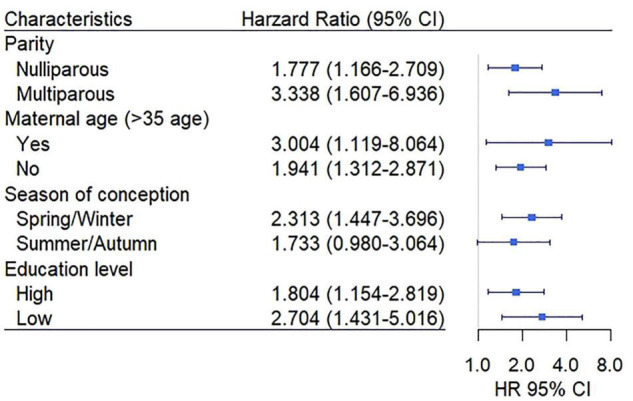
The association between an early GWG category (EwtGCat) and HDP under different subgroup. The Class 1 and Class 2 were defined as low EwtGCat, and Class 3 and Class 4 were grouped into high EwtGCat. The low education level represents high school or below and the high education level includes vocational college and university or above.

The stratified analyses were conducted to assess the effect of EwtGCat on HDP under different variables. The Class 1 and Class 2 of EwtGCat were defined as low EwtGCat and the Class 3 and Class 4 were defined as high EwtGCat. The Figure 3 showed that high EwtGCat was elevated the risk of HDP especially in the women with multipara, maternal age above 35, spring/winter conception and low education level (*HR* = 3.338, 95% *CI*: 1.607–6.936; *HR* = 3.004, 95% *CI*: 1.119–8.064; *HR* = 2.313, 95% *CI*: 1.447–3.696; *HR* = 2.704, 95% *CI*: 1.431–5.016, respectively). In the sensitivity analyses, we failed to observe a significant association between _*T*1_GWG and onset time of HDP (*p* = 0.677).

### Predictive Performance of Early Gestational Weight Gain Category for Hypertensive Disorders of Pregnancy

**FIGURE 4:**
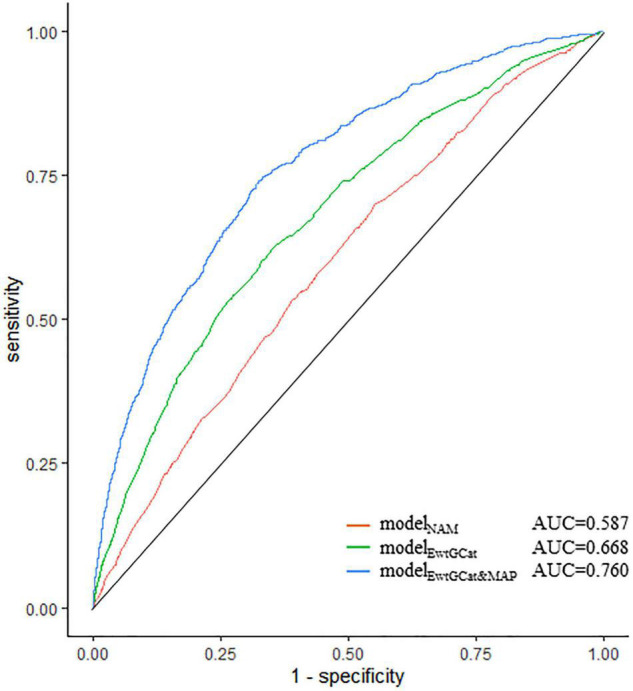
The predictive capacity of prediction models for HDP. The ROC plot demonstrated the predictive capacity of prediction models. The model_*NAM*_, model_*EwtGCat*_, and model_*EwtGCat&MAP*_ were presented in red, green, and blue curves, respectively. The model_*NAM*_ included NAM criteria; model_*EwtGCat*_ included EwtGCat, model_*EwtGCat&MAP*_ was consisted of EwtGCat, MAP_13 *week*_, MAP_2 *week*_. All the models were adjusted by race, age, level of education, employment condition, maternal age (>35 age), gestational season.

We assessed the predictive capacity for HDP by model_*NAM*_, modele_*EwtGCat*_, and model_*EwtGCat&MAP*_. As shown in Figure 4, the AUC, sensitivity, and specificity of the model_*NAM*_, which included NAM criteria and covariables, were 0.587 (95% *CI*: 0.561–0.611), 0.544 (95% *CI*: 0.463–0.606), and 0.578 (95% *CI*: 0.492–0.667). In terms of model_*EwtGCat*_, the AUC was improved to 0.668 (95% *CI*: 0.649–0.688). The model_*EwtGCat&MAP*_ demonstrated the best predictive performance for HDP comparing to the other two model (*AUC* = 0.760, 95% *CI*: 0.739–0.777; sensitivity = 0.703, 95% *CI*: 0.677–0.719; specificity = 0.686, 95% *CI*: 0.635–0.726). The detail information of the predictive models was listed in Supplementary Table 2.

## Discussion

In this study, we observed a significant association between _*T*1_GWG and HDP. The MAP_13 *week*_ and MAP_20 *week*_ mediated the association between _*T*1_GWG and HDP. Meanwhile, we established a risk-specific EwtGCat to assess the risk of HDP according to the pre-pregnant BMI class and _*T*1_GWG of pregnant women. The combination of EwtGCat and MAP showed remarkable greater predictive capacity for HDP in comparison with NAM criteria only.

The previous studies mainly focus on the relationship between total GWG and adverse outcome during the total gestation (17–19). There are few studies investigating the effect of GWG during the first trimester on HDP. And the current tools such as NAM criteria did not access the GWG elevation during the first trimester in terms of HDP (20, 21). The GWG recommendation of NAM criteria provided a different recommended range of GWG per each pre-pregnant BMI class. According to the cut-off points from the NAM criteria, several studies had demonstrated an association between total GWG and HDP (22–24). However, edema and fluid retention that commonly occurred during later trimesters can potentially confound the accurate effect of GWG on HDP (25). The NAM guideline was conducted to reduce the risk of multiple adverse outcomes (26), which failed to distinguish the risk of HDP during this period. In contrast, EGC were constructed by evaluating the risk of HDP per pre-pregnant BMI class. Our study constructed a novel specific-risk category to assess the effect of GWG on the risk of *de novo* HDP and discovered that excessive GWG elevated the risk of HDP in the first trimester, which filled the research gap in the early gestation period.

Abnormal elevated blood pressure was the core symptom of HDP. Numerous studies had demonstrated that the elevated blood pressure associated with the risk of HDP during pregnancy (27–29). However, there were few studies to explore the effect of _*T*1_GWG on blood pressure before the HDP diagnosis. To our knowledge, our study is the first study to assess the serial mediation effects of blood pressure on the association between _*T*1_GWG and HDP before 20 weeks. Our mediation analyses indicated that the serial mediation effects were significant in the total participants and overweight/obesity pregnancies. Thus, the blood pressure before 20 weeks was an independent mediator involving in the association between _*T*1_GWG and HDP. Furthermore, we compared the capacities of EwtGCat and NAM criteria to predict the risk of HDP. The combination of EwtGCat, MAP_13 *week*_, and MAP_20 *week*_ showed significantly higher capacity to predict HDP, compared to the NAM during the first trimester.

Hypertensive disorders of pregnancy is one of the most common gynecological diseases that affects 10% of pregnancies (30). An epidemiological study showed that 10–16% of maternal mortality worldwide was attributed to HDP and this disease was also associated with both a short- and long-term substantial disease burden (31, 32). Increasing number of studies supported that endothelial damage, vascular inflammation, and metabolic dysfunction participate the development of HDP, such as endothelial pathway, NF-κb signaling pathway, abnormal glucose-metabolism, and dyslipidemia (33–36). However, the early identification of HDP remains significantly limited in the clinical practice. GWG is a potential clinical indicator for the risk of HDP, which is non-intrusive and routinely measured in clinical practice. During the first trimester, weight gain came mainly from fat accumulation, while weight of the fetus, extravascular fluid, and maternal fat contributed to weight gain in the later trimesters. Our study focused on the weight gain during the first trimester, which was less likely to be affected by the above concern.

The potential mechanism underlying the association between _*T*1_GWG and HDP remained elusive. In the previous studies, maternal obesity was considered as an important risk factor for HDP. A multicenter Chinese retrospective study showed that overweight and obesity were a risk factor for HDP (37). Another Japanese study obtained a similar result that obese pregnant women were significantly associated with an increased risk of HDP (38). Current theory believes that obesity is a chronic inflammation and accumulating studies have found abnormal immune cells and cytokines in pregnant women with obesity such as CD4 + T cells, macrophages, IL-6, and TNF-α (39–42). Endothelial damage and vascular inflammation are the underlining pathological modifications at every stage of HDP development.

Some strengths were presented in this study. First, the gestational weight gain by the end of first trimester was used to study the temporal relationship between GWG and *de novo* HDP. Second, we constructed a risk-specific EwtGCat which showed greater capacity to identify the risk of *de novo* HDP. Third, this is the first study to explore the mediated mechanism underlying the association between _*T*1_GWG and HDP. Fourth, we illustrated a great potential for using EwtGCat and MAP for the prediction of HDP risk. Meanwhile, there are some limitations to our present study. First, the study subjects were all from the Tongzhou Maternal and Child Health Hospital (Beijing, China), which represent the northern Chinese population. Second, we did not collect the information of maternal lifestyle, such as the physical exercise and stress condition. These factors may be the potential confounding bias on the present study.

## Conclusion

The GWG during the first trimester was associated with the risk of *de novo* HDP. MAP_13 *week*_ and MAP_20 *week*_ partially mediated the association between _*T*1_GWG and HDP. Early GWG category showed a better predictive capacity for the risk of HDP compared to the NAM criteria for first-trimester GWG. Therefore, the pregnancies were supposed to keep the gestational weight gain in an appropriated range to avoid the hazards of HDP. The overweight and obese women especially need to pay more attention on their blood pressure during pregnancy.

## Data Availability Statement

The raw data supporting the conclusions of this article will be made available by the authors, without undue reservation.

## Ethics Statement

The studies involving human participants were reviewed and approved by the Institutional Review Board of Peking University Health Science Center. The patients/participants provided their written informed consent to participate in this study.

## Author Contributions

YJ and ZY: conceptualization, formal analysis, and methodology. ZY, JC, YP, YJ, SZ, HB, HW, SL, JY, and JL: data curation. H-JW: funding acquisition and supervision. ZY, JC, YP, YJ, SZ, HB, HW, and SL: investigation. H-JW, NH, and TS: project administration. ZY: visualization and writing – original draft. YJ and H-JW: writing – review and editing. All authors contributed to the article and approved the submitted version.

## Conflict of Interest

The authors declare that the research was conducted in the absence of any commercial or financial relationships that could be construed as a potential conflict of interest.

## Publisher’s Note

All claims expressed in this article are solely those of the authors and do not necessarily represent those of their affiliated organizations, or those of the publisher, the editors and the reviewers. Any product that may be evaluated in this article, or claim that may be made by its manufacturer, is not guaranteed or endorsed by the publisher.
