# Supplementary material for: The First-Trimester Gestational Weight Gain Associated With de novo Hypertensive Disorders During Pregnancy: Mediated by Mean Arterial Pressure
Source: Front Nutr. 2022 Apr 13;9:862323. doi: 10.3389/fnut.2022.862323 (PMC9045728; doi:10.3389/fnut.2022.862323)
Supplement: Supplementary file 1 [file Table_1.doc]

| **Table S1. Hazard ratios for the associations of gestational weight gain and HDP with different BMI class** | | | | | | |
| --- | --- | --- | --- | --- | --- | --- |
| BMI class | Dummy variables | No HDP (N) | HDP (N) | HR | 95% CI | P value |
| Underweight pregnancies | _T1_GWG≤0 kg |  |  |  |  |  |
|  | No | 1302 | 625 | ref |  |  |
|  | Yes | 29 | 16 | 1.12 | 0.61-2.06 | 0.723 |
|  | 0< _T1_GWG≤1 kg |  |  |  |  |  |
|  | No | 1527 | 400 | ref |  |  |
|  | Yes | 38 | 7 | 0.73 | 0.33-1.64 | 0.444 |
|  | 1< _T1_GWG≤2.5 kg |  |  |  |  |  |
|  | No | 1456 | 471 | ref |  |  |
|  | Yes | 33 | 12 | 1.15 | 0.59-2.22 | 0.684 |
|  | _T1_GWG>2.5 kg |  |  |  |  |  |
|  | No | 1496 | 431 | ref |  |  |
|  | Yes | 35 | 10 | 0.97 | 0.48-1.96 | 0.938 |
| Normal weight pregnancies | _T1_GWG≤0 kg |  |  |  |  |  |
|  | No | 6256 | 3340 | ref |  |  |
|  | Yes | 249 | 121 | 0.89 | 0.71-1.10 | 0.282 |
|  | 0< _T1_GWG≤1 kg |  |  |  |  |  |
|  | No | 7672 | 1924 | ref |  |  |
|  | Yes | 292 | 78 | 1.08 | 0.84-1.39 | 0.547 |
|  | 1< _T1_GWG≤2.5 kg |  |  |  |  |  |
|  | No | 7591 | 2005 | ref |  |  |
|  | Yes | 287 | 83 | 1.09 | 0.86-1.39 | 0.482 |
|  | _T1_GWG>2.5 kg |  |  |  |  |  |
|  | No | 7276 | 2327 | ref |  |  |
|  | Yes | 282 | 88 | 1.00 | 0.78-1.27 | 0.977 |
| Overweight pregnancies | _T1_GWG≤0 kg |  |  |  |  |  |
|  | No | 2826 | 1664 | ref |  |  |
|  | Yes | 271 | 120 | 0.75 | 0.60-0.92 | 0.007 |
|  | 0< _T1_GWG≤1 kg |  |  |  |  |  |
|  | No | 3702 | 788 | ref |  |  |
|  | Yes | 329 | 62 | 1.08 | 0.67-1.16 | 0.368 |
|  | 1< _T1_GWG≤2.5 kg |  |  |  |  |  |
|  | No | 3605 | 885 | ref |  |  |
|  | Yes | 308 | 83 | 1.13 | 0.89-1.44 | 0.328 |
|  | _T1_GWG>2.5 kg |  |  |  |  |  |
|  | No | 3337 | 1153 | ref |  |  |
|  | Yes | 265 | 126 | 1.33 | 1.10-1.68 | 0.004 |
| Obesity pregnancies | _T1_GWG≤0 kg |  |  |  |  |  |
|  | No | 511 | 426 | ref |  |  |
|  | Yes | 104 | 41 | 0.48 | 0.34-0.69 | <0.001 |
|  | 0< _T1_GWG≤1 kg |  |  |  |  |  |
|  | No | 778 | 159 | ref |  |  |
|  | Yes | 111 | 34 | 1.42 | 0.96-2.08 | 0.076 |
|  | 1< _T1_GWG≤2.5 kg |  |  |  |  |  |
|  | No | 788 | 149 | ref |  |  |
|  | Yes | 115 | 30 | 1.37 | 0.91-2.04 | 0.129 |
|  | _T1_GWG>2.5 kg |  |  |  |  |  |
|  | No | 734 | 203 | ref |  |  |
|  | Yes | 105 | 40 | 1.411 | 0.98-2.032 | 0.064 |
| _T1_GWG: the first trimester gestational weight gain; HDP: hypertensive disorders of pregnancy; HR: hazard ratios; CI: confidence interval. The HR was adjusted by race, age, education level, maternal age (>35 age), employment condition, parity. | | | | | | |
