# Supplementary material for: The First-Trimester Gestational Weight Gain Associated With de novo Hypertensive Disorders During Pregnancy: Mediated by Mean Arterial Pressure
Source: Front Nutr. 2022 Apr 13;9:862323. doi: 10.3389/fnut.2022.862323 (PMC9045728; doi:10.3389/fnut.2022.862323)
Supplement: Supplementary file 2 [file Table_2.DOC]

| **Table S2 the distribution of AUC, sensitivity, and specificity for predictive models** | | | | | | | |
| --- | --- | --- | --- | --- | --- | --- | --- |
| Character | Model | Distribution | | | | | |
|  |  | Minimum | P_25_ | median | mean | P_75_ | Maximum |
| AUC | Model_NAM_ | 0.557 | 0.580 | 0.588 | 0.587 | 0.596 | 0.622 |
|  | Model_EwtGCat_ | 0.637 | 0.661 | 0.669 | 0.669 | 0.675 | 0.705 |
|  | Model_EwtGCat&MAP_ | 0.733 | 0.751 | 0.760 | 0.759 | 0.766 | 0.788 |
| Sensitivity | Model_NAM_ | 0.460 | 0.524 | 0.549 | 0.544 | 0.572 | 0.632 |
|  | Model_EwtGCat_ | 0.609 | 0.658 | 0.668 | 0.671 | 0.687 | 0.720 |
|  | Model_EwtGCat&MAP_ | 0.675 | 0.696 | 0.703 | 0.703 | 0.711 | 0.728 |
| Specificity | Model_NAM_ | 0.457 | 0.545 | 0.577 | 0.578 | 0.607 | 0.680 |
|  | Model_EwtGCat_ | 0.479 | 0.556 | 0.577 | 0.578 | 0.599 | 0.655 |
|  | Model_EwtGCat&MAP_ | 0.622 | 0.666 | 0.686 | 0.684 | 0.703 | 0.743 |
| AUC: area under curve; Model_NAM_ included NAM criteria; Model_EwtGCat_ included EwtGCat; Model_EwtGCat&MAP_ was consisted of EwtGCat, MAP_13week_, MAP_20week_. All the models were adjusted by race, age, level of education, employment condition, maternal age (>35 age), conceptional season. | | | | | | | |
